# Thiol/disulfide homeostasis and ischemia modified albumin levels in autoimmune gastritis and their relations with gastric emptying

**DOI:** 10.3906/sag-1902-17

**Published:** 2020-02-13

**Authors:** Emra ASFUROĞLU KALKAN, Serap BOZ, Özcan EREL, Salim NEŞELİOĞLU, Çağdaş KALKAN, İrfan SOYKAN

**Affiliations:** 1 Department of Internal Medicine, Faculty of Medicine, Ankara Numune Education and Research Hospital, Ankara Turkey; 2 Department of Internal Medicine, Faculty of Medicine, Ankara University, Ankara Turkey; 3 Department of Biochemistry, School of Medicine, Yıldırım Beyazıt University, Ankara Turkey; 4 Department of Gastroenterology, Ankara Numune Education and Research Hospital, Ankara Turkey; 5 Department of Gastroenterology, School of Medicine, Ankara University, Ankara Turkey

**Keywords:** Autoimmune gastritis, thiol, disulfide, gastric emptying, oxidative stress

## Abstract

**Background/aim:**

Autoimmune gastritis is an autoimmune and inflammatory disorder. The aim of this study is to examine dynamic thiol/disulfide homeostasis and ischemia modified albumin levels, and to analyze the association between thiol/disulfide homeostasis and gastric emptying time in autoimmune gastritis.

**Materials and methods:**

Thiol/disulfide homeostasis tests and ischemia modified albumin levels were determined in 50 autoimmune gastritis patients and 53 healthy subjects. Patients with delayed and normal gastric emptying were compared by thiol/disulfide homeostasis tests.

**Results:**

The results showed that native thiol (μmol/L), total thiol (μmol/L), and native thiol/total thiol ratio (%) of the patients with autoimmune gastritis decreased compared to the control group (177.7 ± 34.18 vs. 245.25 ± 33.83, P = 0.001, 227.25 ± 36.78 vs. 284.20 ± 27.19, P = 0.03, and 8.84 ± 1.1 vs. 7.74% ± 1.3%, P = 0.001). In addition, native thiol (μmol/L), total thiol (μmol/L), and native thiol/total thiol ratio (%) were found to be lower in patients with delayed gastric emptying (198.65 ± 24.27 vs. 167.12 ± 20.51, 241.81 ± 27.14 vs. 213.92 ± 26.35, 8.34 ± 1.29 vs. 7.20 ± 1.83, P = 0.001). Disulfide level, disulfide/native thiol, disulfide/total thiol (P = 0.001) ratios, and ischemia modified albumin levels (ABSU, 0.71 ± 0.08 vs. 0.83 ± 0.07) were found to be higher in autoimmune gastritis patients with delayed gastric emptying (P = 0.001).

**Conclusion:**

The results showed that thiol/disulfide homeostasis in patients with autoimmune gastritis caused an increase in ischemia modified albumin and disulfide whereas a decrease in thiols. An altered thiol/disulfide balance was also observed in patients with delayed gastric emptying. These results suggest that the oxidative process is involved in patients with autoimmune gastritis.

## 1. Introduction

Autoimmune gastritis (AIG) is an autoimmune disorder. It mainly consists of chronic infiltration of the corpus mucosa of the stomach. It is marked by the reduction or absence of parietal cells and autoantibodies against H+-K+ ATPase [1]. Some studies in the literature show that oxidant radicals increase secondary to inflammation in some autoimmune and autoinflammatory disorders [2–4]. Reactive oxygen species (ROS) can produce molecules leading to cellular damage. The increase in ROS may react with cellular macromolecules and causes lipid peroxidation and nucleic acid damages [5]. Reactive oxygen species induce oxidation of disulfide groups into amino acids containing sulfur. This process is one of the first markers of protein oxidation [6]. 

Thiols are able to react with free radicals in order to provide a defense mechanism against tissue damage [7]. Oxygen molecules oxidize thiol groups of proteins, leading to reversible conversion into disulfide bonds[8]. Thiols form some products due to oxidative stress [9]. These disulfide bonds may be converted into thiol groups once again. A distortion in this homeostasis system may cause different disorders due to the antioxidant protection characteristic of thiol groups [10,11]. Ischemia modified albumin (IMA) is produced as a result of oxidative stress and could be used as an oxidative stress marker [12]. Ischemia modified albumin levels increase in conditions such as tissue damage caused by free radicals [13]. The hypothesis of the study is abnormal thiol/disulfide homeostasis (TDH) and alteration of IMA level may have a place in the pathogenesis of this disorder. Direct measurement of thiol-disulfide levels with a new and automated method is already available [14]. It has been reported that there is a significant relationship between autonomic dysfunction and elevated oxidative stress in diseases such as hypertension and in patients with diabetic peripheric neuropathy [15–17]. Moreover, it has been found that there is a change in autonomic nerve function of some AIG patients. This has revealed a close association between altered autonomic nervous system function and delay in gastric emptying (GE) [18]. Therefore, the aim of our research was to examine dynamic TDH and IMA levels in AIG and specify possible factors associated with this oxidation. We also examined the association between TDH and GE time in AIG as one of the causes of delayed GE [19]. 

## 2. Materials and methods

### 2.1. Patients

The study is a prospective single-center research including 50 AIG patients and 53 healthy individuals. The diagnostic criteria for AIG are: the presence of antiparietal u2918, elevated blood u291b levels and the presence of histology suggesting chronic AIG, including intestinal u291c, pseudopyloric metaplasia, or atrophy of the u291d or body [1]. Subjects with concomitant disorders that may influence TDH [2,20–23], atherosclerotic disorders, diabetes mellitus, kidney disorders, thyroid and liver diseases, malignancy, rheumatic disorders, systemic or other dermatologic diseases, acute or chronic pancreatitis, psychiatric disorders, autoimmune disorders, patients using antioxidant or antilipid agents, and tobacco and alcohol users were excluded from the study. The control group was selected from subjects who were admitted for screening and check-up purposes. TDH parameters and IMA levels were compared between patients and the control group and between patients with delayed and normal GE. The relationships between serum gastrin and chromogranin A levels and TDH, and IMA levels were analyzed.

### 2.2. TDH and ischemia modified albumin

The blood samples were drawn in the fasting state from patients and healthy subjects for the measurement of biochemical parameters and TDH tests. The blood samples were centrifuged for 10 min at 1500 rpm, and the serum was separated. The serum samples were stored at a temperature of −80 °C. TDH tests were carried out as developed by Erel et al. [14]. Briefly, disulfide concentrations were computed as half of the difference between levels of total thiol and native thiol. Then, the disulfide/total thiol percent ratio, disulfide/native thiol percent ratio, and native thiol/total thiol percent ratio were computed [7]. Ischemia modified albumin was determined using a colorimetric cobalt-albumin binding assay as previously described [24]. 

### 2.3. Gastric emptying study 

Gastric emptying time was measured using a 2-hour scintigraphic method [19]. In brief, subjects consumed an isotope-labeled (55 MBq Tc - 99 m macroaggregated albumin) scrambled egg, white meal of 300 kcal. A GE half-time (GET ½) of longer than 110 min was accepted as delayed GE [25]. The TDH and IMA levels were compared between the patients with AIG and the control group. The factors that might affect these parameters were determined. Patients were further stratified into 2 groups: patients with normal GE and patients with delayed GE. Then these 2 groups were analyzed to see whether an abnormality in TDH had any effect on gastric emptying time. The study was approved by the local ethical committee of the related institution and informed consent was obtained from all subjects before conducting the study. Some of the data included in this research were used in other studies previously [18,19].

### 2.4. Statistics

Statistical analysis was performed by using SPSS 16.0 (SPSS, Chicago, IL, USA) for Windows. Results were expressed as percentage of the patients or mean ± SD where appropriate. The Shapiro–Wilk test was used to test the normality of the data, and, depending on the results, parametric or nonparametric tests were selected. Analyses were performed using paired Student’s t-test, Mann–Whitney U test, and Pearson and Spearman correlation tests where appropriate. A P-value of < 0.05 was considered significant. The standard deviation was found to be 0.3 and 0.4 for the 53 patients and 50 healthy subjects, respectively, with a type I error of α = 0.05 and β = 0.20. The power of this study was calculated as 86% (Power Analysis Statistical System 11.0, NCSS Statistical Software, Kaysville, UT, USA).

## 3. Results

**Figure 1:**
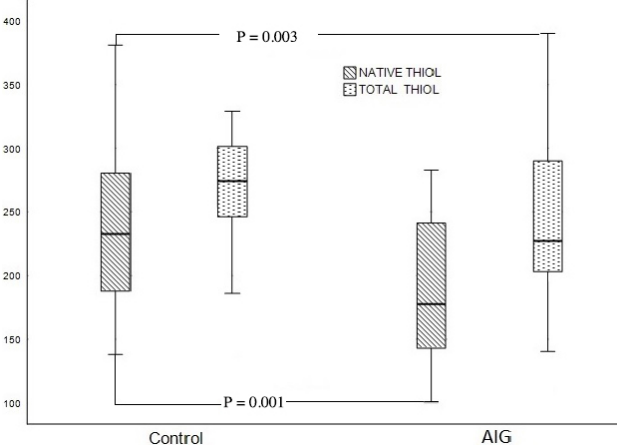
These plots show mean values of serum native thiol (μmol/L) and total thiol (μmol/L) in patients with AIG and the control group. Differences of serum native thiol (P = 0.001), and total thiol (P = 0.03) levels between AIG and control groups were statistically significant. The native thiol and total thiol levels of the patients with AIG decreased compared to the control group (AIG: autoimmune gastritis).

**Figure 2:**
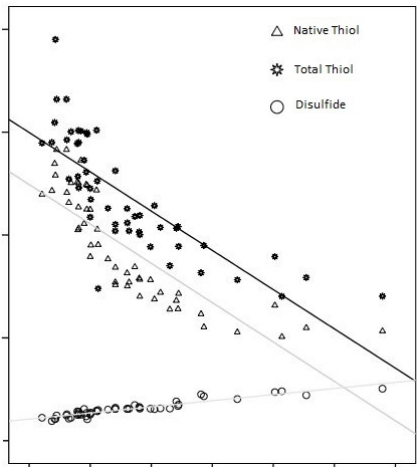
Correlations between GE and disulfide level and native thiol and total thiol levels. While there was a positive correlation between GE and disulfide level, negative correlations were found between GE and native and total thiol levels (GE: gastric emptying).

A total of 50 patients (29 women, mean age 61.3 ± 8.17 years) with AIG and 53 healthy subjects (31 women, 59.5 ± 6.18 years, P = 0.443) were included in the study. It was found that the native thiol (μmol/L), total thiol (μmol/L) and native thiol/total thiol ratio (%) of the patients with AIG decreased compared to the control group (177.7 ± 34.18 vs. 245.25 ± 33.83, P = 0.001, 227.25 ± 36.78 vs. 284.20 ± 27.19, P = 0.03, and 8.84 ± 1.1 vs. 7.74 ± 1.3%, P = 0.001, respectively, Figure 1). Disulfide, disulfide/native thiol, disulfide/total thiol ratios, and IMA of the patients with AIG were found to be higher when compared to the control group (Table 1). Of the 50 patients with AIG, 26 (52%) showed delayed GE and 24 showed normal GE (GET ½: 152.61 ± 26.8   vs, 90.5 ± 6.61 min, P < 0.001). The native thiol (μmol/L), total thiol (μmol/L), and native thiol/ total thiol ratio (%) were found to be lower in the AIG patients with delayed GE than in the patients with normal GE (P = 0.001) (Table 2). The disulfide level, the disulfide/ native thiol, disulfide/total thiol (P = 0.001) ratios, and IMA level were found to be higher in the AIG patients with delayed GE when compared to the patients with normal GE (P = 0.001). The correlation analysis between TDH tests and other parameters within the AIG patients are shown in detail in Tables 3 and 4. Positive correlations between disulfide, disulfide/native thiol, disulfide/total thiol, serum gastrin, and chromogranin A levels were found. However, correlation analysis revealed a negative correlation between native thiol, total thiol, native thiol/total thiol, IMA, serum gastrin, and chromogranin A levels. While there was a positive correlation between GE and disulfide level, we found negative correlations between GE and native and total thiol levels (Figure 2). 

**Table 1:** TDH parameters in patients with autoimmune gastritis and control group.

Variables	Control (n = 53)	AIG (n = 50)	P
Native thiol (μmol/L) (mean ± SD)	245.25 ± 33.83	177.7 ± 34.18	0.001
Total thiol (μmol/L) (mean ± SD)	284.20 ± 27.19	227.25 ± 36.78	0.003
Disulfide (μmol/L) (mean ± SD)	25.37 ± 2.27	32.45 ± 4.43	0.002
Disulfide/native thiol (%)	10.31 ± 2.7	16.37 ± 1.05	0.001
Disulfide/total thiol (%)	8.06 ± 2.3	12.8 ± 2.07	0.002
Native thiol/total thiol (%)	8.84 ± 1.1	7.74 ± 1.3	0.001
IMA (ABSU)	0.61 ± 0.07	0.77 ± 0.05	0.001

(AIG: autoimmune gastritis, ABSU: absorbance unit)

**Table 2:** TDH parameters among patients with delayed gastric emptying and normal GE.

Variables	AIG (GET ½ < 110)(n = 24)	AIG (GET ½ ≥ 110)(n = 26)	P
Native thiol (μmol/L) (mean ± SD)	198.65 ± 24.27	167.12 ± 20.51	0.001
Total thiol (μmol/L) (mean ± SD)	241.81 ± 27.14	213.92 ± 26.35	0.001
Disulfide (μmol/L) (mean ± SD)	30.49 ± 4.42	34.41 ± 3.79	0.001
Disulfide/native thiol (%)	13.28 ± 2.36	20.29 ± 1.94	0.001
Disulfide/total thiol (%)	9.73 ± 2.28	16.13 ± 1.66	0.001
Native thiol/total thiol (%)	8.34 ± 1.29	7.20 ± 1.83	0.001
IMA (ABSU)	0.71 ± 0.08	0.83 ± 0.07	0.001

(AIG: Autoimmune gastritis, GET ½: Gastric emptying half time, ABSU: Absorbance unit)

**Table 3:** The correlation analysis of TDH parameters and other risk factors in the autoimmune gastritis patients.

	Disulfide/native thiol		Disulfide/total thiol		Native thiol/total thiol		IMA	
Variables	r	P	r	P	r	P	r	P
Gastrin (ng/L)	0.681	0.005	0.745	0.001	–0.662	0.009	–0.565	0.007
Chromogranin A (μg/L)	0.764	0.002	0.866	0.002	–0.642	0.006	–0.612	0.003

**Table 4:** The correlation analysis of TDH parameters and other risk factors in the autoimmune gastritis patients.

	Native thiol		Total thiol		Disulfide	
Variables	r	P	r	P	r	P
Gastrin (ng/L)	–0.757	0.001	–0.612	0.003	0.657	0.003
Chromogranin A (μg/L)	–0.644	0.007	–0.628	0.001	0.675	0.001

## 4. Discussion

It has been revealed that the native thiol, total thiol, and native thiol/total thiol ratio of AIG patients significantly decreased when compared to the control group, and disulfide, disulfide/native thiol, disulfide/total thiol ratios, and IMA of AIG patients were found to be significantly higher when compared to the control group. The TDH protects human body from oxidative stress, and this balance plays a pivotal role in detoxification and antioxidant protection. Provided that disulfide formation increases, functional and structural alterations are seen in most of the systems. This condition has an adverse impact on protection against oxidative stress [26,27]. Although the investigation of this subject is a matter of debate, various studies have been conducted using this method, including inflammatory bowel diseases [26,27], diabetes mellitus [28], and cardiovascular diseases [29], and a close relation with oxidative stress has been found. Ates et al. showed that altered TDH in subclinical hypothyroidism and thyroid autoantibodies was positively correlated with thiol oxidation [2]. Although the data in the literature regarding the role of oxidative stress in the development of AIG is not sufficient. It is known that there is a greater increase in oxidant radicals than in antioxidant molecules leading to oxidative stress in autoimmune thyroid diseases, which are organ-specific autoimmune disorders similar to AIG [30–32]. Excessive production of reactive oxygen species and a deranged redox state are accepted as some of the pathogenic mechanisms underlying systemic autoimmune response. The increase in the level of reactive oxygen species may cause oxidative alteration of lipids, proteins, and carbohydrates. This oxidative alteration of proteins leads to pathogenic antibodies in autoimmune diseases [33]. Baser et al. examined the oxidative status of autoimmune thyroiditis patients by measurement of total antioxidant status, total oxidant status, and IMA, and found that oxidants increased, while antioxidants decreased, in patients with euthyroid autoimmune thyroiditis. They concluded that increased oxidative stress may play a role in autoimmune thyroid disorders [32]. Kaplan et al. studied TDH in 73 patients with gluten-sensitive enteropathy, a chronic autoimmune disease, by the same method [34]. They found an altered TDH in patients with gluten-sensitive enteropathy compared to healthy subjects, and concluded that this alteration was associated with autoimmunity and inflammation. Kalkan et al. showed that native thiol and total thiol levels were significantly higher in patients with lichen planus, an autoimmune inflammatory disease of the mucocutaneous tissue [20]. Koseoglu et al. showed that disulfide/total thiol percent ratios and disulfide/native thiol percent ratios were significantly higher in patients with acute pancreatitis, whereas the total and native thiol levels and native thiol/total thiol percent ratio were significantly lower. These changes indicate that the thiol/disulfide redox balance shifted to the disulfide bond side in acute pancreatitis [23].

Patients with AIG exhibited altered autonomic function, indicating an important association between autonomic dysfunction and delayed GE [18]. This result and the existence of a positive association between elevated oxidative stress and autonomic dysfunction have led us to examine the relationship between GE and TDH [35,36]. The native thiol, total thiol, and native thiol / total thiol ratio were found to be lower in AIG patients with delayed GE than in patients with normal GE. The disulfide level, the disulfide/native thiol, and disulfide/total thiol ratios were found to be higher in AIG patients with delayed GE than in patients with normal GE. In this regard, altered TDH may cause delayed GE due to autonomic nerve dysfunction. We also examined IMA levels of patients and of the control group. IMA is a modified form of albumin and may be used as an indicator of oxidative stress [37]. IMA is produced as a consequence of changes in albumin’s capacity in order to bind heavy metals. It is widely used to evaluate myocardial ischemia. However, the increase in IMA levels is also observed in disorders such as obesity, type 2 diabetes mellitus, hypercholesterolaemia, psoriasis, and familial Mediterranean fever, which are associated with oxidative stress [38–40]. It has been suggested that IMA may have a role as an oxidative stress marker. In our study, higher levels of IMA were found in the AIG patients than the healthy controls. Kucuk et al. studied IMA levels in FMF patients with an autoinflammatory disease and found that IMA levels were higher in the familial Mediterranean fever group than in healthy controls [41]. Furthermore, Capkin et al. observed that IMA levels were higher in patients with Bechet’s disease, which is an inflammatory disease similar to familial Mediterranean fever, than in the control group [42]. Our study found that IMA levels were higher in patients with delayed GE than in patients with normal GE. Our results also showed a significant inverse association between IMA and serum gastrin, and chromogranin A levels. It has been reported that essential oils and their secondary metabolites are related as potent antioxidants and free radical scavengers in chronic inflammation [43]. Furthermore, some products, such as synthetic trans-Δ9-tetrahydrocannabinol dissolved in sesame oil, have proven to possess a potential antioxidative effect in inflammatory arthritis. Therefore, AIG patients with altered TDH may benefit from agents with antioxidative properties [44].

In conclusion, our study has revealed that TDH was altered and IMA levels increased in patients with AIG when compared to healthy controls. Furthermore, the dynamic TDH shifted through disulfide form more in AIG patients with delayed GE than in patients with normal GE. Altered TDH observed in these patients may shed light on the pathophysiology of this disorder and could suggest therapeutic options, such as antioxidant agents in the management of AIG.
